# The British Medical Association

**Published:** 1873-10-01

**Authors:** 


### Medical Examiner.

*A Semi-Monthly Journal of Medical Sciences.*

EDITED BY

N. S. DAVIS, M. D., AND F. H. DAVIS, M. D.

Chicago, Oct. 1st, 1873.

### EDITORIAL.

### THE BRITISH MEDICAL ASSOCIATION.

The forty-first annual meeting of this Association was held at King's College, London, commencing August 5th, 1873.

The address on Medicine was delivered by Prof. E. A. Parkes, F.R.S., of the Army Med. School; the address on Physiology by Dr. Burdon Sanderson; and the address on Surgery by Prof. John Wood, of King's College. An abstract of the latter address we publish in the present number of The Examiner.

A London journal, *The Doctor*, in commenting editorially on the success of the meeting, says:

"The celebration of the forty-first annual meeting of the British Medical Association at King's College last month has been called by some of the lay journals a Medical Congress, and deserves the name. It is pronounced by our contemporaries to have been a success; but most of them seem to think more of the amusements and the "feeding" than of the scientific part of the programme.

"As a reunion of Medical men "on pleasure bent," we may look on it as successful; but we feel some regret that such a gathering should not elicit more solid advantages. King's College was full of people eager to join in excursions, to meet old acquaintances, to look at or speak to the notabilities, to listen to a *portion* of one of the addresses. But the sections were almost deserted, save by those who had papers to read, or who desired to speak.

"The division of the work into sections is in reality a failure. Half-a-dozen meetings going on at the same time cannot be all well attended. Then a fair proportion of those who would work in the sections were desirous of attending two or more, and in the effort to catch the papers they wanted to hear, missed all. This was very unfortunate, and destroyed the enthusiasm of several of our acquaintances.

"The addresses are always a strong feature. That of the President this year was very genial and homely, and offered a cordial welcome to all; the beginning and end of it were therefore welcome; and Sir William Ferguson was as popular as ever. In the central part of his address, however, he attacked the water question, for which he has been in various papers attacked in turn. He took what may be called a common-sense view, in opposition to the chemical notion; but the world being as it is, we prefer the chemical view, just as we prefer pure to impure water. Sir William, as a surgeon, is unequalled; as a chemist, we can scarcely accept his authority."

It appears from the above quotations that a portion of the English medical press are inclined to criticise the proceedings of the British Association in a good deal the same spirit that some of our American journals have taken up the workings of our national organization.

